# Implant success in grafted vs non-grafted posterior mandibular sites: A clinical study

**DOI:** 10.6026/973206300213253

**Published:** 2025-09-30

**Authors:** Preeti B. Astagi, Harikrishnan Balachandran Pillai, Kolli Giri, Suresh Chinnakutti, Bharathiram Guduri, Prateek Tripathy, Rahul Tiwari

**Affiliations:** 1 Department of Prosthodontics Crown and Bridge, Maratha Mandal's NGH Institute of Dental Sciences, Bauxite Road, Belgaum, Rajiv Gandhi University of Health Sciences, Bangalore, Karnataka, India; 2 Department of Periodontology, Azeezia College of Dental Sciences and Research, Kollam, Kerala, India; 3 Department of Oral and Maxillofacial Surgery, Siddhartha Institute of Dental Sciences, Chinnaoutpalli, Gannavaram, Andhra Pradesh, India; 4 Department of Oral and Maxillofacial Surgery, Vinayaka Mission's Sankarachariyar Dental College, Vinayaka Mission's Research Foundation (Deemed to be University), Salem, Tamil Nadu, India; 5 Department of Oral and Maxillofacial Surgery, GSL Dental College, Rajahmundry, Andhra Pradesh, India; 6 Department of Oral and Maxillofacial Surgery, Hi-Tech Dental College and Hospital, Bhubaneswar, Odisha, India; 7 Department of Oral and Maxillofacial Surgery, RKDF Dental College and Research Centre, Sarvepalli Radhakrishnan University, Bhopal, Madhya Pradesh, India

**Keywords:** Dental implants, alveolar bone grafting, mandible, treatment outcome, survival rate

## Abstract

Implant success in grafted and non-grafted posterior mandibular sites is always a topic of discussion for decades. A retrospective review of 80 patients and 120 implants was conducted. Clinical and radiographic success was evaluated after three years. Implant survival was slightly higher in non-grafted sites (96.7%) than grafted sites (93.3%). Marginal bone loss was greater in grafted sites. Thus, we compare implant success in grafted and non-grafted posterior mandibular sites.

## Background:

Implant placement in the posterior mandible presents unique biomechanical and anatomical challenges [1]. Limited bone height and quality, especially after tooth loss, often necessitate bone grafting to achieve primary stability [2]. While bone augmentation techniques such as autogenous block graft and guided bone regeneration have expanded the indications for implant therapy, questions remain regarding the long-term outcomes of implants in grafted versus non-grafted sites [3]. Several retrospective and prospective studies have reported high survival rates of implants in both conditions, typically exceeding 90% [4, 5]. However, evidence also suggests that grafted sites may demonstrate greater marginal bone loss due to remodeling processes [6]. Posterior mandibular bone, characterized by dense cortical architecture, often provides favorable conditions for primary stability and long-term osseointegration [7]. Nonetheless, in cases with significant resorption, grafting remains the standard approach to restore ridge volume [8]. The success of implants is not determined solely by survival rates but also by functional integration, peri-implant bone stability, and absence of complications such as infection or implant mobility [9, 10]. Despite advances, clinical comparisons between grafted and non-grafted posterior mandibles remain limited, warranting further investigation. Therefore, it is of interest to compare implant success in grafted and non-grafted posterior mandibular sites.

## Materials and Methods:

This retrospective clinical study analyzed patient records with dental implants placed in posterior mandibular sites. A total of 120 implants in 80 patients (mean age: 46 ± 9 years; 42 males, 38 females) were included. Implants were divided into two groups:

[1] Group A (Grafted): 60 implants placed in sites reconstructed with autogenous or xenograft materials.

[2] Group B (Non-grafted): 60 implants placed in native bone without grafting.

## Inclusion criteria:

Systemically healthy patients, single or multiple implants placed in posterior mandibular sites, minimum of 3 years follow-up. Exclusion criteria: uncontrolled systemic disease, smoking >10 cigarettes/day, and parafunctional habits.

## Clinical parameters recorded:

Implant mobility, peri-implant probing depth (PPD), and bleeding on probing (BOP). Radiographic evaluation assessed marginal bone loss (MBL) on standardized periapical radiographs. Implant success was defined as absence of pain, infection, mobility, or radiolucency, with MBL <1.5 mm in the first year and <0.2 mm annually thereafter.

## Statistical analysis:

Chi-square test compared survival rates between groups; independent t-test analyzed mean bone loss. Significance was set at p < 0.05.

## Results:

A total of 120 implants were evaluated after 3 years. Overall survival rate was 95%.

[1] Group A (Grafted): 56/60 implants survived (93.3%).

[2] Group B (Non-grafted): 58/60 implants survived (96.7%) Table 1 (see PDF).

Marginal bone loss was significantly greater in grafted sites (mean 1.35 ± 0.4 mm) compared with non-grafted sites (0.92 ± 0.3 mm) (p < 0.05). No statistically significant difference in peri-implant probing depths or BOP was observed Table 2 (see PDF).

## Discussion:

This clinical study compared implant outcomes in grafted and non-grafted posterior mandibular sites, demonstrating overall high success rates exceeding 90%. These results are consistent with previous evidence indicating implant survival in grafted sites is comparable to non-grafted bone [11]. Although survival rates were slightly lower in grafted sites (93.3% vs 96.7%), the difference was not statistically significant, aligning with studies reporting no significant survival discrepancies between grafted and native posterior mandibular bone [12, 13]. However, grafted sites exhibited greater marginal bone loss, which concurs with earlier findings that graft remodeling contributes to higher resorption risk [14, 15]. The posterior mandible typically provides dense cortical bone advantageous for stability, which may explain the slightly better outcomes in non-grafted sites [16]. Nonetheless, grafting remains indispensable in cases with significant atrophy, where achieving ideal implant positioning and stability is otherwise impossible [17]. Peri-implant probing depth and soft tissue health were comparable between groups, supporting the notion that proper surgical protocol and maintenance care mitigate differences in peri-implant tissue response [18]. Importantly, both groups exhibited success rates above the threshold of 85%, widely considered a clinical benchmark for implant therapy [19]. Limitations include the retrospective design, modest sample size and absence of longer follow-up beyond 3 years. Future randomized controlled trials with larger cohorts and longer observation periods are required to validate these findings and explore patient-reported outcomes [20]. In conclusion, implants placed in both grafted and non-grafted posterior mandibular sites achieve high success rates, though marginal bone loss is greater in grafted bone. Clinical decision-making should weigh the necessity of grafting against the potential for slightly increased resorption.

## Conclusion:

Implant success in posterior mandibular sites is high in both grafted and non-grafted conditions. While grafting shows slightly more bone loss, both approaches yield predictable clinical outcomes.

## References

[1] Lorenz J (2025). Clin Oral Implants Res..

[2] Memenga-Nicksch S (2025). J Dent..

[3] Gurbanov E, Plugmann P (2024). Oral Health Prev Dent..

[4] Kupka J.R (2024). Clin Oral Investig..

[5] Liu Y (2024). Clin Oral Implants Res..

[6] Romito G.A (2024). J Clin Periodontol..

[7] Romito G.A (2023). Clin Oral Implants Res..

[8] Romito G.A (2022). J Clin Periodontol..

[9] McKenna G (2022). J Evid Based Dent Pract..

[10] Deng C (2022). Br J Oral Maxillofac Surg..

[11] Chatelet C (2022). J Stomatol Oral Maxillofac Surg..

[12] Mahardawi A (2023). J Dent..

[13] Lin Y (2023). BMC Oral Health..

[14] Khoury F, Hanser T (2022). Int J Oral Implantol (Berl)..

[15] Cucchi A (2024). Clin Oral Implants Res..

[16] Barausse C (2024). Clin Oral Implants Res..

[17] Al Haydar B (2023). Int J Oral Maxillofac Implants..

[18] Zhang Y (2024). BMC Oral Health..

[19] Pabst A.M (2025). J Clin Med..

[20] Debortoli C (2024). J Clin Med..

